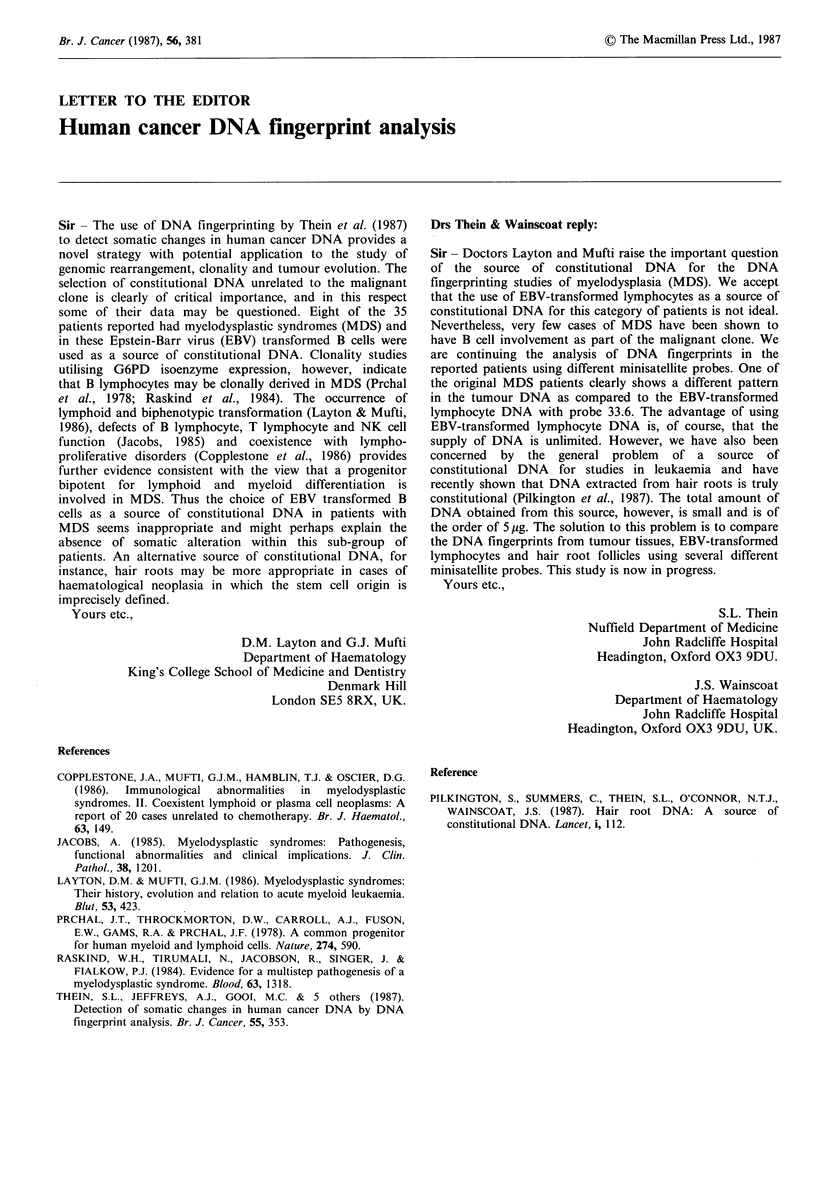# Human cancer DNA fingerprint analysis.

**DOI:** 10.1038/bjc.1987.208

**Published:** 1987-09

**Authors:** D. M. Layton, G. J. Mufti


*Br. J. Cancer* (1987), **56**, 381 © The Macmillan Press Ltd., 1987

### LETTER TO THE EDITOR

### Human cancer DNA fingerprint analysis

**Sir** – The use of DNA fingerprinting by Thein *et al.* (1987) to detect somatic changes in human cancer DNA provides a novel strategy with potential application to the study of genomic rearrangement, clonality and tumour evolution. The selection of constitutional DNA unrelated to the malignant clone is clearly of critical importance, and in this respect some of their data may be questioned. Eight of the 35 patients reported had myelodysplastic syndromes (MDS) and in these Epstein-Barr virus (EBV) transformed B cells were used as a source of constitutional DNA. Clonality studies utilising G6PD isoenzyme expression, however, indicate that B lymphocytes may be clonally derived in MDS (Prchal *et al.*, 1978; Raskind *et al.*, 1984). The occurrence of lymphoid and biphenotypic transformation (Layton & Mufti, 1986), defects of B lymphocyte, T lymphocyte and NK cell function (Jacobs, 1985) and coexistence with lymphoproliferative disorders (Copplestone *et al.*, 1986) provides further evidence consistent with the view that a progenitor bipotent for lymphoid and myeloid differentiation is involved in MDS. Thus the choice of EBV transformed B cells as a source of constitutional DNA in patients with MDS seems inappropriate and might perhaps explain the absence of somatic alteration within this sub-group of patients. An alternative source of constitutional DNA, for instance, hair roots may be more appropriate in cases of haematological neoplasia in which the stem cell origin is imprecisely defined.

Yours etc.,

D.M. Layton and G.J. Mufti
Department of Haematology
King's College School of Medicine and Dentistry
Denmark Hill
London SE5 8RX, UK.

### References

COPPLESTONE, J.A., MUFTI, G.J.M., HAMBLIN, T.J. & OSCIER, D.G. (1986). Immunological abnormalities in myelodysplastic syndromes. II. Coexistent lymphoid or plasma cell neoplasms: A report of 20 cases unrelated to chemotherapy. *Br. J. Haematol.*, **63**, 149.

JACOBS, A. (1985). Myelodysplastic syndromes: Pathogenesis, functional abnormalities and clinical implications. *J. Clin. Pathol.*, **38**, 1201.

LAYTON, D.M. & MUFTI, G.J.M. (1986). Myelodysplastic syndromes: Their history, evolution and relation to acute myeloid leukaemia. *Blut*, **53**, 423.

PRCHAL, J.T., THROCKMORTON, D.W., CARROLL, A.J., FUSON, E.W., GAMS, R.A. & PRCHAL, J.F. (1978). A common progenitor for human myeloid and lymphoid cells. *Nature*, **274**, 590.

RASKIND, W.H., TIRUMALI, N., JACOBSON, R., SINGER, J. & FIALKOW, P.J. (1984). Evidence for a multistep pathogenesis of a myelodysplastic syndrome. *Blood*, **63**, 1318.

THEIN, S.L., JEFFREYS, A.J., GOOI, M.C. & 5 others (1987). Detection of somatic changes in human cancer DNA by DNA fingerprint analysis. *Br. J. Cancer*, **55**, 353.